# The genome sequence of the European turtle dove, *Streptopelia turtur* Linnaeus 1758

**DOI:** 10.12688/wellcomeopenres.17060.1

**Published:** 2021-07-27

**Authors:** Jenny C. Dunn, Keith C. Hamer, Antony J. Morris, Philip V. Grice, Michelle Smith, Craig Corton, Karen Oliver, Jason Skelton, Emma Betteridge, Jale Dolucan, Michael A. Quail, Shane A. McCarthy, Marcela Uliano-Silva, Kerstin Howe, James Torrance, William Chow, Sarah Pelan, Ying Sims, Richard Challis, Jonathan Threlfall, Daniel Mead, Mark Blaxter

**Affiliations:** 1 University of Lincoln, Lincoln, LN6 7TS, UK; 2 School of Biology, University of Leeds, Leeds, LS2 9JT, UK; 3 Centre for Conservation Science, Royal Society for the Protection of Birds, Sandy, Bedfordshire, SG19 2DL, UK; 4 Natural England, Peterborough, PE1 1NG, UK; 5 Wellcome Sanger Institute, Hinxton, Cambridgeshire, CB10 1SA, UK; 6 Achilles Therapeutics plc, London, W6 8PW, UK; 7 Department of Genetics, University of Cambrudge, Cambridge, CB2 3EH, UK; 8 Owlstone Medical, Cambridge, CB4 0GJ, UK

**Keywords:** Streptopelia turtur, European turtle dove, genome sequence, chromosomal

## Abstract

We present a genome assembly from an individual female *Streptopelia turtur* (the European turtle dove; Chordata; Aves; Columbidae). The genome sequence is 1.18 gigabases in span. The majority of the assembly is scaffolded into 35 chromosomal pseudomolecules, with the W and Z sex chromosomes assembled.

## Species taxonomy

Eukaryota; Metazoa; Chordata; Aves; Columbiformes; Columbidae; Streptopelia; *Streptopelia turtur* Linnaeus 1758 (NCBI:txid177155).

## Introduction

The European turtle dove, *Streptopelia turtur*, breeds throughout Europe, Central Asia, the Middle East and North Africa, overwintering in north Sub-Saharan Africa. Populations in the Atlantic archipelago of Britain and Ireland are primarily located in southern and eastern England. *S. turtur* populations are in rapid decline in the UK, having fallen by 98% between 1970 and 2018, making them critically endangered; they are also vulnerable to global extinction ( Burns *et al.*, 2020). Several causes have been put forward for this collapse in population. Changes in farming practices and agricultural intensification in the UK have reduced the availability of wild plant seeds, increasing the reliance of *S. turtur* on anthropogenic seed sources ( Browne & Aebischer, 2003); a negative association between nestling condition and consumption of seeds from anthropogenic sources has been reported, although this association was positive for adult birds ( Dunn *et al.,* 2018). Additionally, infection with the protozoan parasite *Trichomonas gallinae* has been identified as a cause of death in adults and nestlings ( Stockdale *et al.,* 2015). The length of breeding seasons and the number of breeding attempts of *S. turtur* have markedly reduced, meaning that fewer young are hatched each year ( Browne & Aebischer, 2004). Large populations of migrating birds are also hunted in Mediterranean countries, such as France, Spain and Morocco, compounding this decline in numbers. The genome sequence described here will be of utility to researchers assessing the vulnerability of *S. turtur* to parasitic infections, and to those interested in population genomics and supporting the numbers of this declining species.

## Genome sequence report

**Figure 1.**
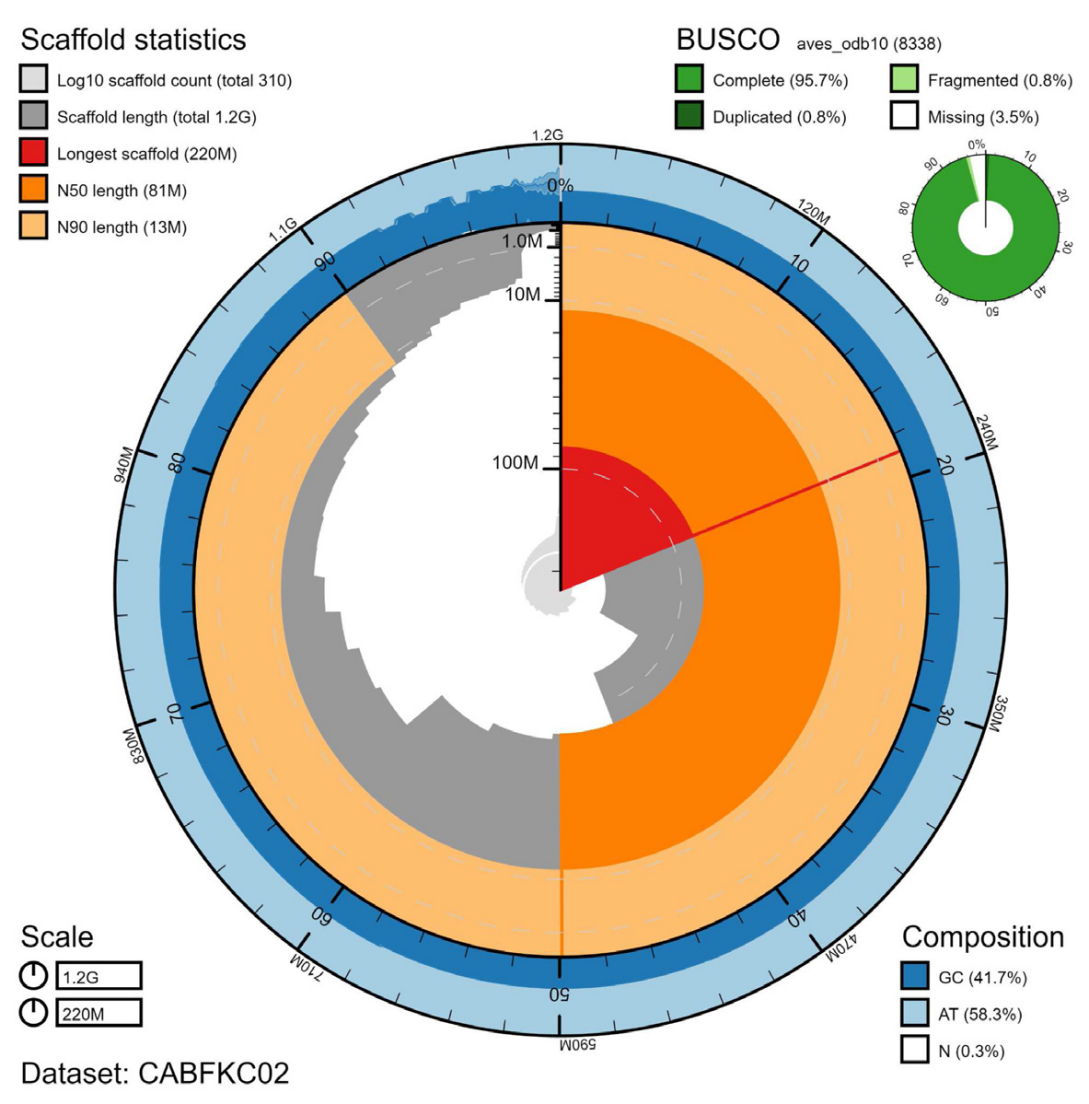
Genome assembly of *Streptopelia turtur*, bStrTur1.2: metrics. The BlobToolKit Snailplot shows N50 metrics and BUSCO gene completeness. An interactive version of this figure is available at https://blobtoolkit.genomehubs.org/view/Streptopelia%20turtur/dataset/CABFKC02/snail.

The genome was sequenced from a blood sample collected from a single live female *S. turtur* during routine population health checks. A total of 34-fold coverage in Pacific Biosciences single-molecule long reads (N50 22 kb) and 45-fold coverage in 10X Genomics read clouds (from molecules with an estimated N50 of 34 kb) were generated. Primary assembly contigs were scaffolded with chromosome conformation Hi-C data. The Hi-C scaffolds were validated using BioNano Genomics long range restriction maps (106-fold effective coverage). Manual assembly curation corrected 54 missings/misjoins and removed 1 haplotypic duplication, reducing the scaffold number by 23.59%, increasing the scaffold N50 by 19.08% and decreasing the assembly length by 0.01%. The final assembly has a total length of 1.18 Gb in 357 sequence scaffolds with a scaffold N50 of 81.4 Mb ( Table 1). The majority, 98.3%, of the assembly sequence was assigned to 35 chromosomal-level scaffolds representing 33 autosomes (numbered by synteny to the chicken, *Gallus gallus* domesticus: GCA_000002315.5), and the W and Z sex chromosomes ( Figure 1– Figure 4; Table 2). The assembly has a BUSCO v5.1.2 ( Simão *et al.,* 2015) completeness of 95.7% using the aves_odb10 reference set. While not fully phased, the assembly deposited is of one haplotype. Contigs corresponding to the second haplotype have also been deposited.

**Table 1.** Genome data for *Streptopelia turtur*, bStrTur1.2.

*Project accession data*		
Assembly identifier	bStrTur1.2
Species	*Streptopelia turtur*
Specimen	bStrTur1
NCBI taxonomy ID	NCBItxid:177155
BioProject	PRJEB32724
BioSample ID	SAMEA994735
Isolate information	Female, blood sample
*Raw data accessions*	
PacificBiosciences SEQUEL I	ERR3041797-ERR3041814
10X Genomics Illumina	ERR3229775, ERR3316037-ERR3316040
Hi-C Illumina	ERR4179379-ERR4179383
BioNano	ERZ1392829
*Genome assembly*	
Assembly accession	GCA_901699155.2
*Accession of alternate haplotype*	GCA_901699165.2
Span (Mb)	1,179
Number of contigs	1,246
Contig N50 length (Mb)	3.79
Number of scaffolds	311
Scaffold N50 length (Mb)	81.4
Longest scaffold (Mb)	222
BUSCO * genome score	C:95.7%[S:94.9%,D:0.8%],F:0.8%,M:3.5%, n:8338

*BUSCO scores based on the aves_odb10 BUSCO set using v5.1.2. C= complete [S= single copy, D=duplicated], F=fragmented, M=missing, n=number of orthologues in comparison. A full set of BUSCO scores is available at https://blobtoolkit.genomehubs.org/view/Streptopelia%20turtur/dataset/CABFKC02/busco.

**Figure 2.**
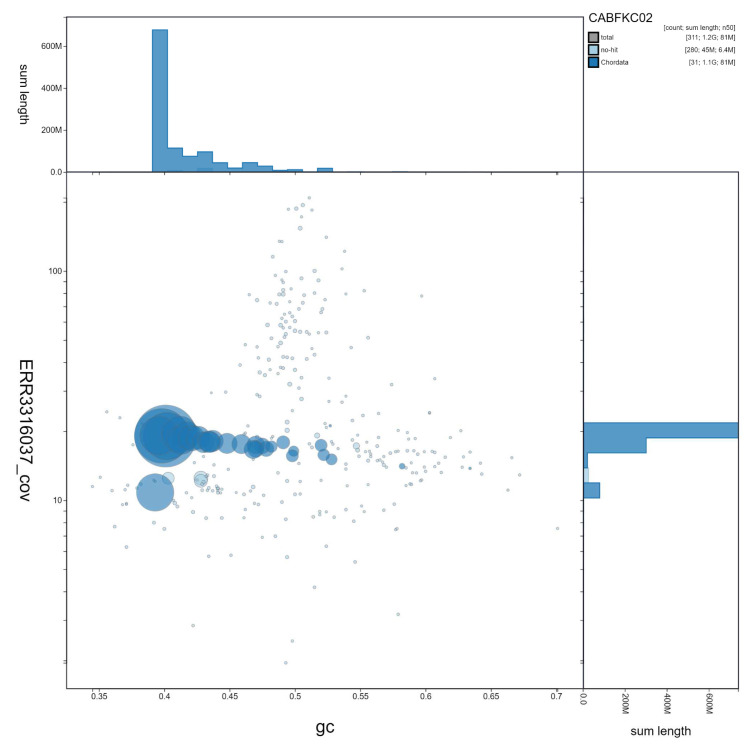
Genome assembly of *Streptopelia turtur*, bStrTur1.2: GC coverage. BlobToolKit GC-coverage plot. An interactive version of this figure is available at https://blobtoolkit.genomehubs.org/view/Streptopelia%20turtur/dataset/CABFKC02/blob.

**Figure 3.**
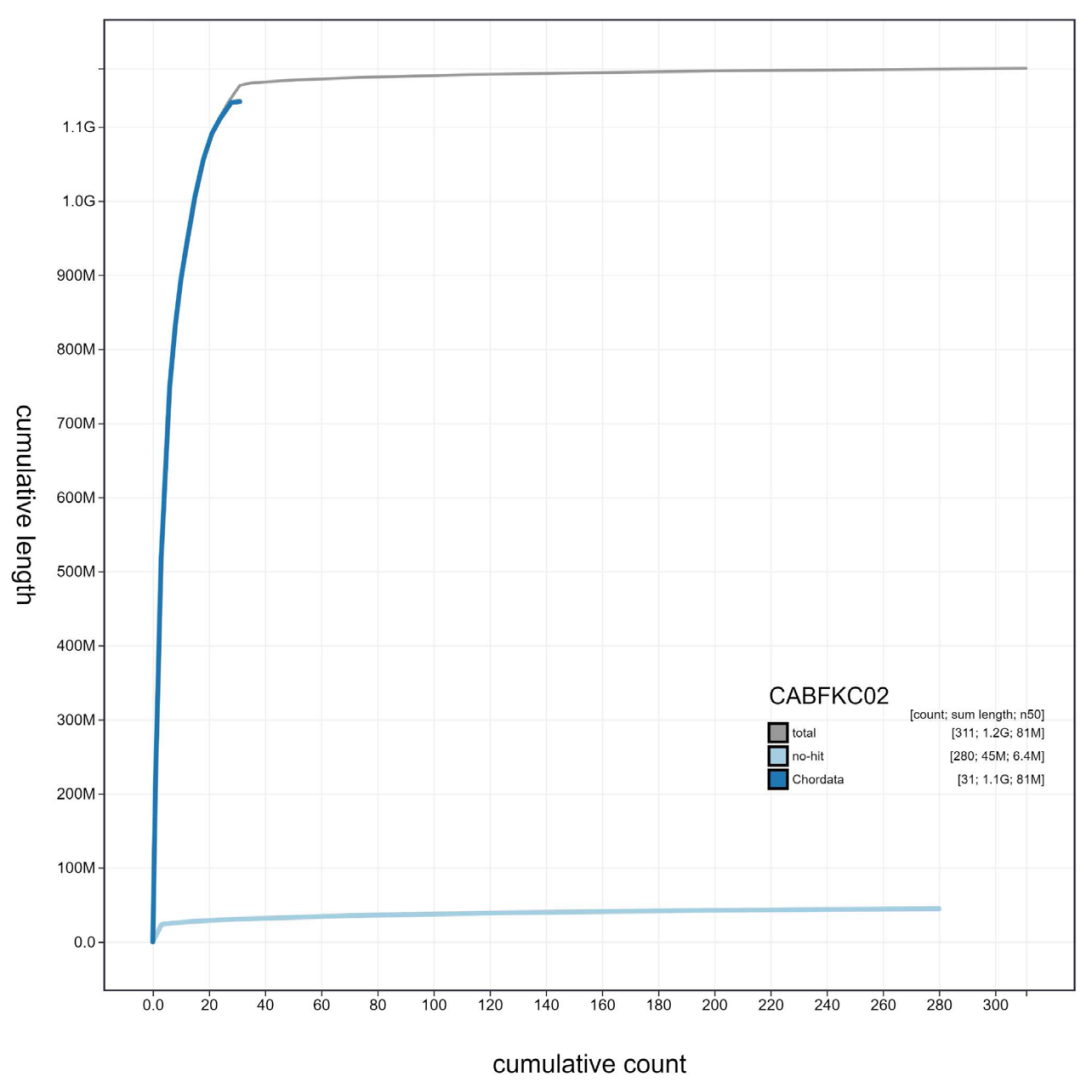
Genome assembly of *Streptopelia turtur*, bStrTur1.2: cumulative sequence. BlobToolKit cumulative sequence plot. An interactive version of this figure is available at https://blobtoolkit.genomehubs.org/view/Streptopelia%20turtur/dataset/CABFKC02/cumulative.

**Figure 4.**
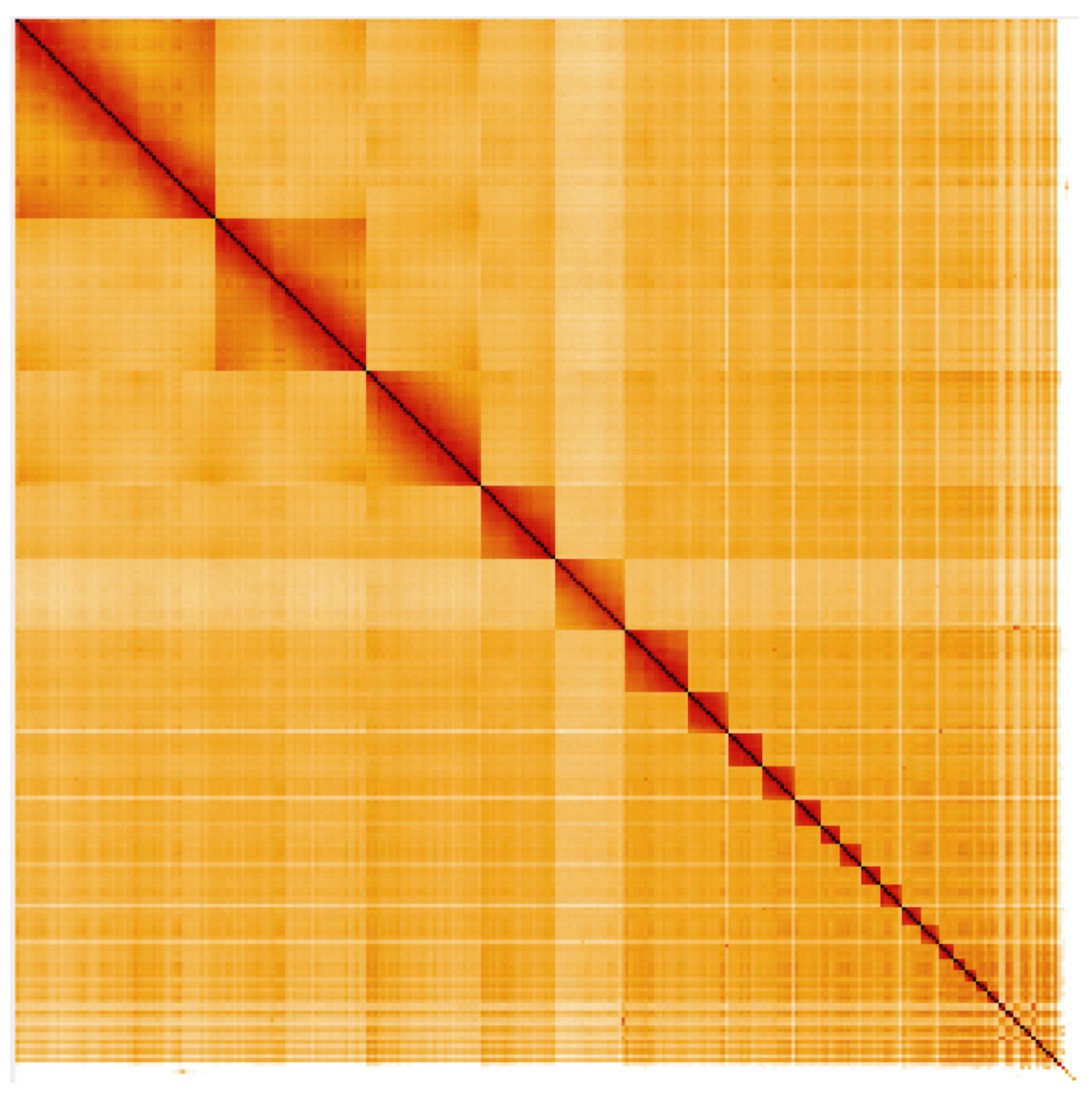
Genome assembly of *Streptopelia turtur*, bStrTur1.2: Hi-C contact map. Hi-C contact map of the bStrTur1 assembly, visualized in HiGlass ( Kerpedjiev *et al.,* 2018).

**Table 2.** Chromosomal pseudomolecules in the genome assembly of *Streptopelia turtur*, bStrTur1.2.

INSDC accession	Chromosome	Size (Mb)	GC%
LR594551.2	1	222.12	0.40
LR594552.1	2	169.96	0.40
LR594553.2	3	126.22	0.40
LR594554.2	4	81.37	0.40
LR594556.1	5	70.85	0.41
LR594558.1	6	40.34	0.42
LR594557.1	7	42.93	0.41
LR594559.1	8	34.57	0.42
LR594560.2	9	27.80	0.43
LR594562.1	10	23.07	0.44
LR594563.1	11	22.26	0.43
LR594561.1	12	23.32	0.43
LR594564.1	13	20.81	0.45
LR594565.1	14	18.48	0.46
LR594567.2	15	15.32	0.47
LR594569.2	17	11.44	0.48
LR594570.2	18	12.59	0.47
LR594568.1	19	10.86	0.48
LR594566.1	20	16.38	0.47
LR594571.2	21	7.81	0.49
LR594577.1	22	4.50	0.50
LR594572.1	23	5.94	0.52
LR594573.1	24	5.82	0.48
LR594579.1	25	1.00	0.58
LR594575.2	26	6.47	0.52
LR594576.2	27	6.49	0.50
LR594574.2	28	5.37	0.53
OU015479.1	29	22.41	0.44
LR594580.2	30	0.71	0.52
LR594581.1	32	0.02	0.53
LR594578.1	33	1.28	0.55
LR594555.2	Z	77.54	0.39
OU015480.1	W	8.84	0.43

## Methods

The European turtle dove specimen was taken from blood collected from a live bird during routine health checks of populations in Marks Tey, Essex, UK (latitude 51.874N, longitude 0.729E; grid reference TL8823). The sample was taken under Home Office (Animals Scientific Procedures Act, ASPA) licence number PPL 7007641); the bird was caught and handled under a British Trust for Ornithology ringing license.

DNA was extracted using an agarose plug extraction from a blood sample following the BioNano Genomics Prep Blood and Cell Culture DNA Isolation Protocol. Pacific Biosciences (PacBio) CLR long read and 10X Genomics read cloud sequencing libraries were constructed according to manufacturers’ instructions. Sequencing was performed by the Scientific Operations core at the Wellcome Sanger Institute on Pacific Biosciences SEQUEL I and Illumina HiSeq X instruments. Ultra-high molecular weight DNA was extracted using the BioNano Genomics Prep Animal Tissue DNA Isolation Soft Tissue Protocol and assessed by pulsed field gel and Qubit 2 fluorimetry. DNA was labeled for BioNano Genomics optical mapping following the BioNano Genomics Prep Direct Label and Stain (DLS) Protocol, and run on one Saphyr Optical Instrument chip flowcell (BioNano Genomics). Hi-C data were generated using the Arima Hi-C kit v1 by Arima Genomics, San Diego, USA, using the Illumina HiSeqX sequencing instrument.

Assembly was carried out following the Vertebrate Genome Project pipeline v1.6 ( Rhie *et al.,* 2020) with Falcon-unzip ( Chin *et al.,* 2016); haplotypic duplication was identified and removed with purge_haplotigs ( Roach *et al.,* 2018) and a first round of scaffolding carried out with 10X Genomics read clouds using scaff10x. Hybrid scaffolding was performed using the BioNano Genomics DLE-1 data and BioNano Solve. Scaffolding with Hi-C data ( Rao *et al.,* 2014) was carried out with SALSA2 ( Ghurye *et al.*, 2019). The Hi-C scaffolded assembly was polished with arrow using the PacBio data, then polished with the 10X Genomics Illumina data by aligning to the assembly with longranger align, calling variants with freebayes ( Garrison & Marth, 2012) and applying homozygous non-reference edits using bcftools consensus. Two rounds of the Illumina polishing were applied. The assembly was checked for contamination and corrected using the gEVAL system ( Chow *et al.,* 2016) as described previously ( Howe *et al.,* 2021). Manual curation was performed using evidence from BioNano Genomics (using the BioNano Access viewer), using HiGlass and Pretext. Figure 1– Figure 3 and BUSCO v5.1.2 scores were generated using BlobToolKit ( Challis *et al.,* 2020). Table 3 gives version numbers of the software tools used in this work.

**Table 3.** Software tools used.

Software tool	Version	Source
Falcon-unzip	falcon-kit 1.1.1	( Chin *et al.,* 2016)
purge_haplotigs	1.0.2	( Roach *et al.,* 2018)
SALSA2	2.2	( Ghurye *et al.,* 2019)
Bionano Solve	3.3_10252018	https://bionanogenomics.com/support/software-downloads/
scaff10x	3.0	https://github.com/wtsi-hpag/Scaff10X
arrow	2.2.2	https://github.com/PacificBiosciences/GenomicConsensus
longranger align	2.2.2	https://support.10xgenomics.com/genome-exome/software/ pipelines/latest/advanced/other-pipelines
freebayes	1.1.0-3-g961e5f3	( Garrison & Marth, 2012)
bcftools consensus	1.9	http://samtools.github.io/bcftools/bcftools.html
HiGlass	1.11.6	( Kerpedjiev *et al.,* 2018)
PretextView	0.0.4	https://github.com/wtsi-hpag/PretextMap
gEVAL	N/A	( Chow *et al.,* 2016)
BlobToolKit	2.6.1	( Challis *et al.,* 2020)

## Data availability

European Nucleotide Archive: Streptopelia turtur (European turtle dove) genome assembly, bStrTur1. Accession number PRJEB32724.

The genome sequence is released openly for reuse. The *S. turtur* genome sequencing initiative is part of the Wellcome Sanger Institute’s “ 25 genomes for 25 years” project. It is also part of the Vertebrate Genome Project (VGP) ordinal references programme and the Darwin Tree of Life (DToL) project. All raw data and the assembly have been deposited in the ENA. The genome will be annotated and presented through the Ensembl pipeline at the European Bioinformatics Institute. Raw data and assembly accession identifiers are reported in Table 1.
