# Supplementary material for: The genome sequence of the European turtle dove, Streptopelia turtur Linnaeus 1758
Source: Wellcome Open Res. 2021 Jul 27;6:191. [Version 1] doi: 10.12688/wellcomeopenres.17060.1 (PMC11252641; doi:10.12688/wellcomeopenres.17060.1)
Supplement: Peer review history

# Reviewer report: Reviewer response for version 1

**DOI:** 10.21956/wellcomeopenres.18842.r88407

**Published:** 2024-07-16

**Authors:** Ruiqi Li

**Affiliations:** 1 University of Colorado Boulder, Boulder, Colorado, USA

Dunn *et al. *presented a high quality genome of the European turtle dove, *Streptopelia turtur*. The genome is of high quality. I only have a few minor comments.

 How does this genome help researchers assess the vulnerability of *S. turtur* to parasitic infections? Could you explain it further?

 Could you please include a photo of the specimen, or at least a photo of the species?

Are sufficient details of methods and materials provided to allow replication by others?

Yes

Is the rationale for creating the dataset(s) clearly described?

Yes

Are the datasets clearly presented in a useable and accessible format?

Yes

Are the protocols appropriate and is the work technically sound?

Yes

Reviewer Expertise:

genomics

I confirm that I have read this submission and believe that I have an appropriate level of expertise to confirm that it is of an acceptable scientific standard.

# Reviewer report: Reviewer response for version 1

**DOI:** 10.21956/wellcomeopenres.18842.r47530

**Published:** 2021-12-21

**Authors:** Noor White

**Affiliations:** 1 Neurobiology Neurodegeneration and Repair Laboratory, National Eye Institute, National Institutes of Health, Bethesda, Maryland, USA

In this article, the authors present a genome assembly of the European turtle dove. The methods are clear and well detailed. The authors use a variety of sequence data types, including several long-read technologies in order to present a solid assembly. The article is clear and straight-forward, and the genome assembly and associated raw data will be of use to the scientific community.

Are sufficient details of methods and materials provided to allow replication by others?

Yes

Is the rationale for creating the dataset(s) clearly described?

Yes

Are the datasets clearly presented in a useable and accessible format?

Yes

Are the protocols appropriate and is the work technically sound?

Yes

Reviewer Expertise:

Evolutionary genetics, ornithology, comparative genomics, retinal cell biology

I confirm that I have read this submission and believe that I have an appropriate level of expertise to confirm that it is of an acceptable scientific standard.
